# PEGylated insulin-like growth factor-I affords protection and facilitates recovery of lost functions post-focal ischemia

**DOI:** 10.1038/s41598-017-00336-z

**Published:** 2017-03-21

**Authors:** Kim Parker, Antonio Berretta, Stefanie Saenger, Manaswini Sivaramakrishnan, Simon A. Shirley, Friedrich Metzger, Andrew N. Clarkson

**Affiliations:** 1 Department of Anatomy and Brain Health Research Center, University of Otago, Dunedin 9054, New Zealand; 2 F. Hoffmann-La Roche Ltd., pRED, Pharma Research & Early Development, Roche Innovation Center Basel, Grenzacherstrasse 124, CH-4070 Basel, Switzerland; 3 Brain Research New Zealand, University of Otago, Dunedin 9054, New Zealand; 4 Faculty of Pharmacy, The University of Sydney, Sydney, Australia

## Abstract

Insulin-like growth factor-I (IGF-I) is involved in the maturation and maintenance of neurons, and impaired IGF-I signaling has been shown to play a role in various neurological diseases including stroke. The aim of the present study was to investigate the efficacy of an optimized IGF-I variant by adding a 40 kDa polyethylene glycol (PEG) chain to IGF-I to form PEG-IGF-I. We show that PEG-IGF-I has a slower clearance which allows for twice-weekly dosing to maintain steady-state serum levels in mice. Using a photothrombotic model of focal stroke, dosing from 3 hrs post-stroke dose-dependently (0.3–1 mg/kg) decreases the volume of infarction and improves motor behavioural function in both young 3-month and aged 22–24 month old mice. Further, PEG-IGF-I treatment increases GFAP expression when given early (3 hrs post-stroke), increases Synaptophysin expression and increases neurogenesis in young and aged. Finally, neurons (P5–6) cultured *in vitro* on reactive astrocytes in the presence of PEG-IGF-I showed an increase in neurite length, indicating that PEG-IGF-I can aid in sprouting of new connections. This data suggests a modulatory role of IGF-I in both protective and regenerative processes, and indicates that therapeutic approaches using PEG-IGF-I should be given early and where the endogenous regenerative potential is still high.

## Introduction

With 15 million people suffering annually, stroke remains a leading cause of mortality and morbidity worldwide. To date, drug therapies have primarily focused on preventing the loss of nerve cells, however, these therapies have failed to translate into the clinic^1^. As a result tissue plasminogen activator (tPA) remains the only FDA approved pharmaceutical for ischemic stroke with a therapeutic window of 4.5–6 hrs^2^. Therefore, novel targets are required for developing much needed therapies to help sufferers of stroke.

With successful recovery following a stroke being hindered by a lack of viable treatment options, stroke patients are limited to physical therapies and neurorehabilitation practices^3^. These therapies utilize the underlying mechanisms of neuroplasticity in the brain that allows for reprogramming of motor functions into spared cortical areas^3^. Research over the past decade has resulted in significant advances in our understanding of mechanisms associated with post-stroke recovery^4–6^. Recent findings have highlighted that modulation of key developmental signaling pathways, such as Ephrin^7^, Sonic hedgehog (Shh)^8^, or Insulin-Like Growth Factor I (IGF-I)^9^, can play a role in reactivating neural connections and improving functional recovery.

IGF-I plays critical roles in neurite outgrowth *in vitro* ^10^ and during cortical development^11^. In addition, IGF-I modulates the cascade of cell death and affords protection against neuronal injury^12–15^. Further, IGF-I has been shown to protect both grey and white matter when given following stroke^16, 17^, but not when given before, further implying a regenerative action^13^. In addition, elevated levels of IGF-I following stroke are associated with improved functional outcomes^18^, suggesting a further role for IGF-I in neural regeneration. However, the role that IGF-I plays during post-stroke repair is poorly understood. A recent study showed that local delivery of IGF-I in a hydrogel injected into the stroke cavity, providing sustained IGF-I release to the peri-infarct cortex for a period of up to 4 weeks, does not induce changes in cortical connections compared to vehicle treated stroke controls^9^.

As IGF-I delivery is limited by a short systemic half-life (approximately 4 hrs in rodents), PEGylated IGF-I was generated via the addition of a 40 kDa polyethylene glycol (PEG) chain to lysine 68 of IGF-I^19, 20^. This optimized form of IGF-I results in decreased renal clearance and provides sustained levels of IGF-I to the periphery and brain^20^, allowing for a dosing regime of once a week in humans (twice-weekly in rodents) to maintain steady-state serum levels^19–21^.

In this study, we sought to investigate whether administration of PEG-IGF-I could be used to improve outcomes of stroke in mice through neuroprotective, and or neuro-regenerative mechanisms. Our primary aim was to assess whether there was a dose-dependent effect of PEG-IGF-I, and at what stage after stroke dosing would be most effective, starting 3 hrs, 1-day or 5-days post-insult. To evaluate efficacy we used an *in vivo* model of focal ischemic stroke^22–24^ and analyzed infarct volume, behavioral functional recovery, as well as the ability of PEG-IGF-I to augment axonal sprouting, gliosis and neurogenesis post-stroke. To further investigate the biological function of PEG-IGF-I signaling on neurite outgrowth after injury, we used an *in vitro* model where astrocytes were traumatized to render them reactive, similar to reactive astrogliosis found *in vivo* after stroke^7, 8^, and evaluated whether PEG-IGF-I may be able to stimulate neurite outgrowth in this injury model.

## Results

### PEG-IGF-I dosing results in a prolonged elevation in serum IGF-I levels

Low IGF-I levels have previously been reported to be a good outcome measure of poor recovery after stroke^25^. Having a dosing regime that would allow serum IGF-I levels to be safey elevated would most likely aid in a better functional outcome for stroke patients. However, dosing with human recombinant IGF-I (rhIGF-I) that has a short half-live (approximately 4 hrs in rodents) results in an undesirable peak-trough dose profile. Prior studies have demonstrated that a single dose of PEG-IGF-I can provide homeostatic serum levels with an increased circulating half-life compared to IGF-I^19, 20^.

**Figure 1:**
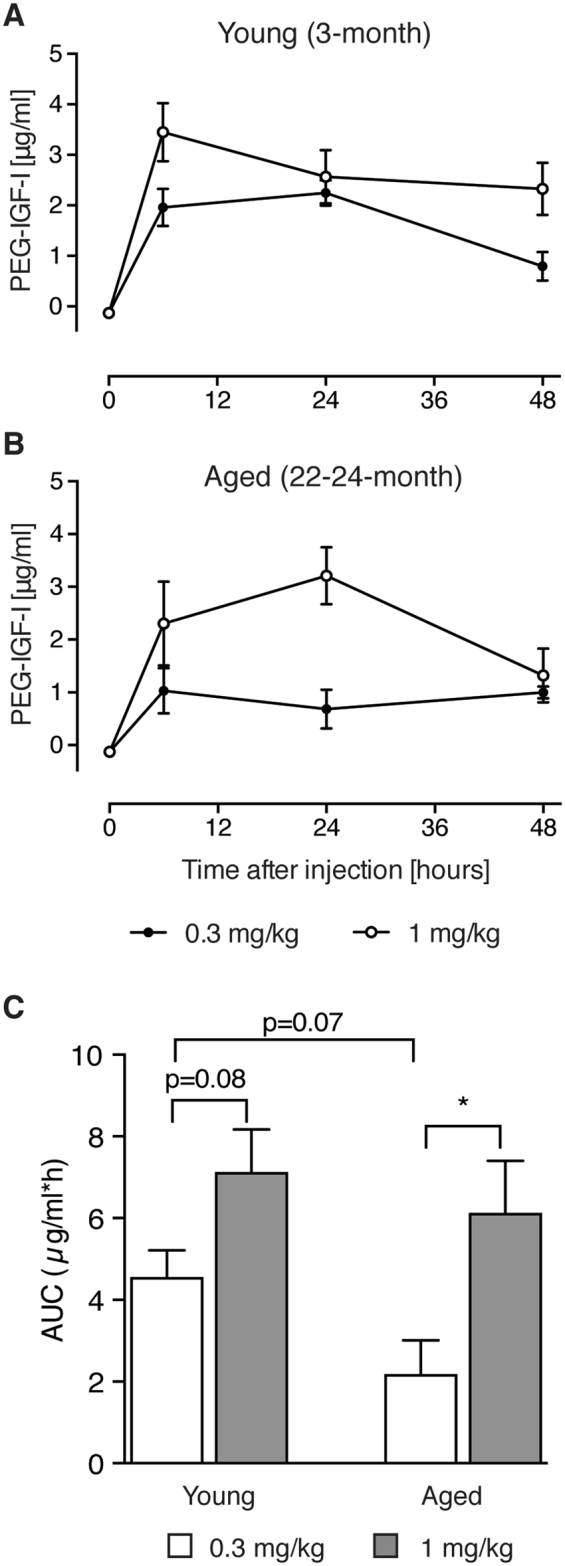
Serum concentrations of PEG-IGF-I were assessed following a single injection of either 0.3 mg/kg, or 1 mg/kg dose in young (**A**) and aged (**B**) mice. Levels could be detected in both young and aged mice even 48 hrs post-injection with levels being slightly higher in the young. (**C**) Areas under curve (AUC, µg/ml*h) of the pharmacokinetic profiles of PEG-IGF-I treatment over 48 hrs. *p < 0.05 by t-test. An n = 4 per dose and time point was used.

To confirm prior studies, we first evaluated serum drug levels at various time points following a single i.p. injection. PEG-IGF-I, dosing at either 0.3 or 1 mg/kg resulted in a sustained elevation, with serum levels of PEG-IGF-I still at 794 ± 282 ng/ml (0.3 mg/kg; n = 4) and 2326 ± 518 ng/ml (1 mg/kg; n = 4) 48 hrs post i.p. dosing in young mice (Fig. 1A). These results are in good agreement with previous findings, showing that PEG-IGF-I has an extended serum half-life as compared to rhIGF-I, allowing for a greater period of time between doses^19, 20^.

As almost 80–90% of clinical strokes occur in people who are over 65 years^26^, it is important that preclinical stroke studies evaluating potential therapies include aged animals. Therefore, we administered PEG-IGF-I to a cohort of old (22–24 months) male mice and assessed serum IGF-I levels, as these have not been measured in aged mice following PEG-IGF-I treatment. Following a single i.p. injection of PEG-IGF-I, dosing at either 0.3 or 1 mg/kg resulted in a sustained elevation, with serum levels of PEG-IGF-I still at 999 ± 112 ng/ml (0.3 mg/kg; n = 4) and 1321 ± 508 ng/ml (1 mg/kg; n = 4) 48 hrs post i.p. dosing (Fig. 1B). Interestingly, serum levels in aged were almost half those found in young, age-related differences in dosing that could be attributed to differences in metabolism.

These data are surprising, as our previous data suggested fully linear distribution in different mouse models^19, 27^ and in humans^21^, with very similar absolute drug levels in the lower ug/ml range for these doses. As IGF-I exerts its biological activity in a fully homeostatic manner, the area under curve (AUC) over the entire 48 hrs time frame defines the biological activity rather than the peaks observed after 6 or 24 hrs. Therefore, we generated AUC data of the PK curves (Fig. 1C). Statistical comparison revealed that trends were observed but due to the low sample size (n = 4) only the aged group comparison showed a significant difference (*P* < 0.05). However, the means of these data clearly confirm the linearity of exposure for the aged group, with 3 times higher AUC for the 1 than the 0.3 mg/kg dose.

### PEG-IGF-I decreases infarct volume after focal cerebral ischemia in young and aged mice

**Figure 2:**
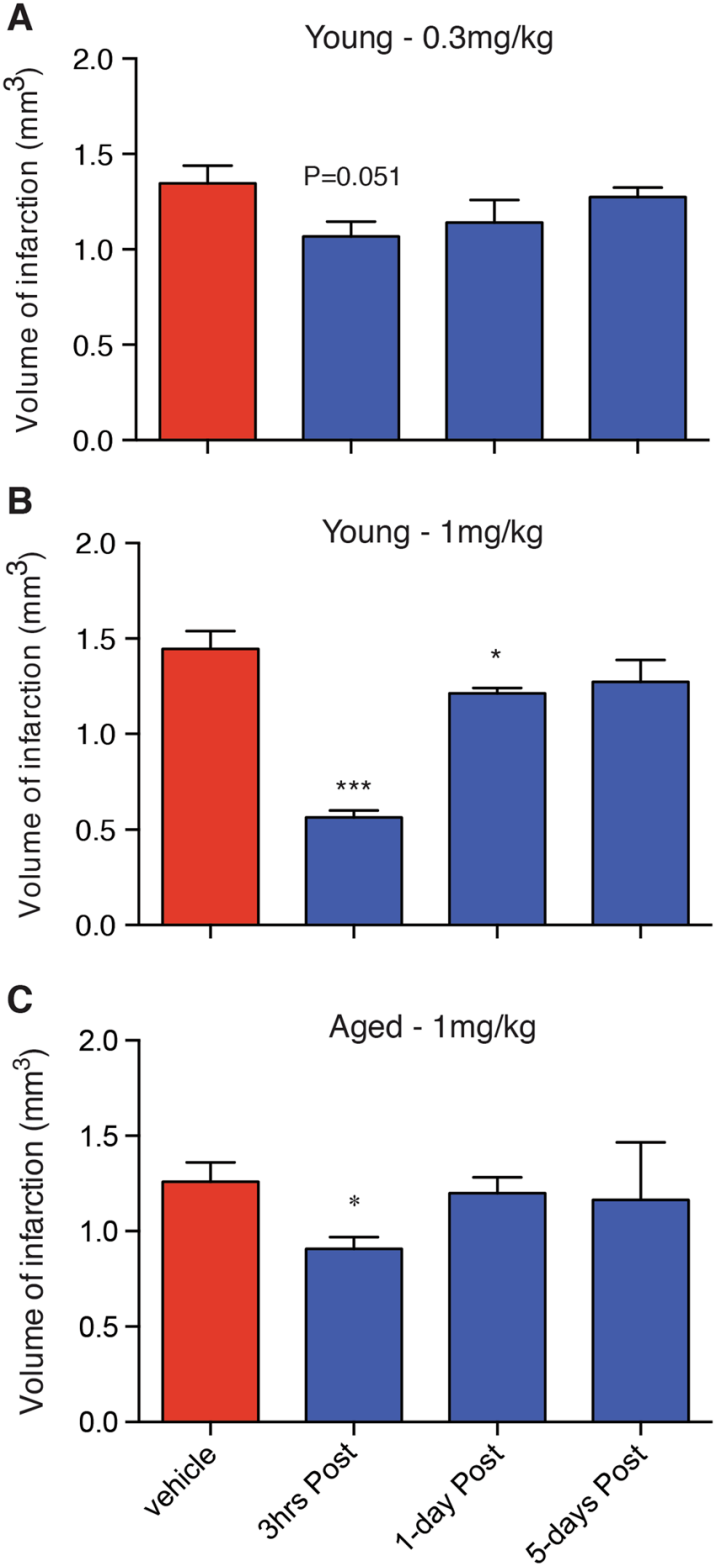
Early PEG-IGF-I treatment affords significant protection in both young and aged mice. Histological assessment was assessed at either 56-days post-stroke in young mice that received 0.3 or 1 mg/kg PEG-IGF-I (**A** and **B** respectively), or 48-days post-stroke in aged mice that received 1 mg/kg PEG-IGF-I (**C**). An n = 5 per group were used. *P < 0.05 and ***P < 0.0001 compared to stroke + vehicle-treated controls.

IGF-I has previously been shown to be protective in different models of hypoxic/ischemic brain injury and stroke^13, 28^. However, as rhIGF-I has a short systemic half-life, the frequency of dosing required to achieve prolonged increases in a safe manner makes this an unlikely treatment option to take into the clinic. Therefore, we sought to investigate whether treatment with PEG-IGF-I, where serum levels are still elevated 48 hrs after a single i.p. injection, has similar protective effects after focal ischemic stroke. To assess the therapeutic potential of PEG-IGF-I, infarct volume was assessed 56-days post-focal ischemia following treatment starting at either 3 hrs, 1-day or 5-days post-stroke (Fig. 2).

Not all strokes have a reperfusion component, hence we investigated the protective effects of PEG-IGF-I in the focal photothrombosis model of stroke that has minimal reperfusion and is traditionally harder to protect against cell death. Further, we have previously reported that the photothrombosis model is an ideal model of stroke for assessing motor functional recovery^24^. Mice were treated with an initial i.p. dose of PEG-IGF-I (0.3 or 1 mg/kg) or PBS vehicle, either 3 hrs (both), or 1-day and 5-days (PEG-IGF-I only) post-stroke, with dosing continued twice-weekly thereafter. Treatment with PEG-IGF-I resulted in both a time and dose-dependent decrease in infarct volume. Dosing at 0.3 mg/kg resulted in a small yet non-significant decrease in infarct volume when dosing started at 3 hrs post-stroke (vehicle, 1.35 ± 0.09 mm^3^ versus 0.3 mg/kg PEG-IGF-I, 1.07 ± 0.08 mm^3^, *P* = 0.051, n = 5 per group; Fig. 2A). No differences in infarct volume were observed following 0.3 mg/kg PEG-IGF-I dosing, starting from 1 or 5-days post-stroke (Fig. 2A). Dosing at 1 mg/kg resulted in a significant decrease in infarct volume when treatment started either 3 hrs or 1-day post-stroke but not when dosing started 5-days post-stroke (vehicle, 1.45 ± 0.09 mm^3^ versus 1 mg/kg PEG-IGF-I (3 hrs), 0.56 ± 0.04 mm^3^, *P* < 0.0001; 1-day, 1.21 ± 0.03 mm^3^, *P* = 0.0429; 5-day, 1.27 ± 0.12 mm^3^, *P* = 0.2726: n = 5 per group; Fig. 2B). These data indicate that giving PEG-IGF-I earlier affords greater protection. However, even delaying the treatment by 24 hrs can result in a decrease in infarct volume in young animals with the right dosing, suggesting that PEG-IGF-I has both protective and regenerative properties.

To further confirm the therapeutic potential of PEG-IGF-I for older patient populations, aged mice were treated with an initial i.p. dose of PEG-IGF-I (1 mg/kg) or vehicle starting either 3 hrs, 1-day or 5-days post-stroke, with dosing continued twice-weekly thereafter. Treatment with PEG-IGF-I resulted in a decrease in infarct volume when treatment started at 3 hrs post-stroke but not when started at either 1 or 5-days post-stroke (vehicle, 1.26 ± 0.11 mm^3^ versus 1 mg/kg PEG-IGF-I (3 hrs), 0.91 ± 0.06 mm^3^, *P* < 0.05; 1-day, 1.19 ± 0.08 mm^3^, *P* = 0.6600; 5-day, 1.16 ± 0.31 mm^3^, *P* = 0.7736: n = 6 per group; Fig. 2C). The degree of protection noted in the aged mice was markedly less than what was observed following treatment in the young mice.

### PEG-IGF-I facilitates an improvement in functional recovery even when given at a delay after focal cerebral ischemia

**Figure 3:**
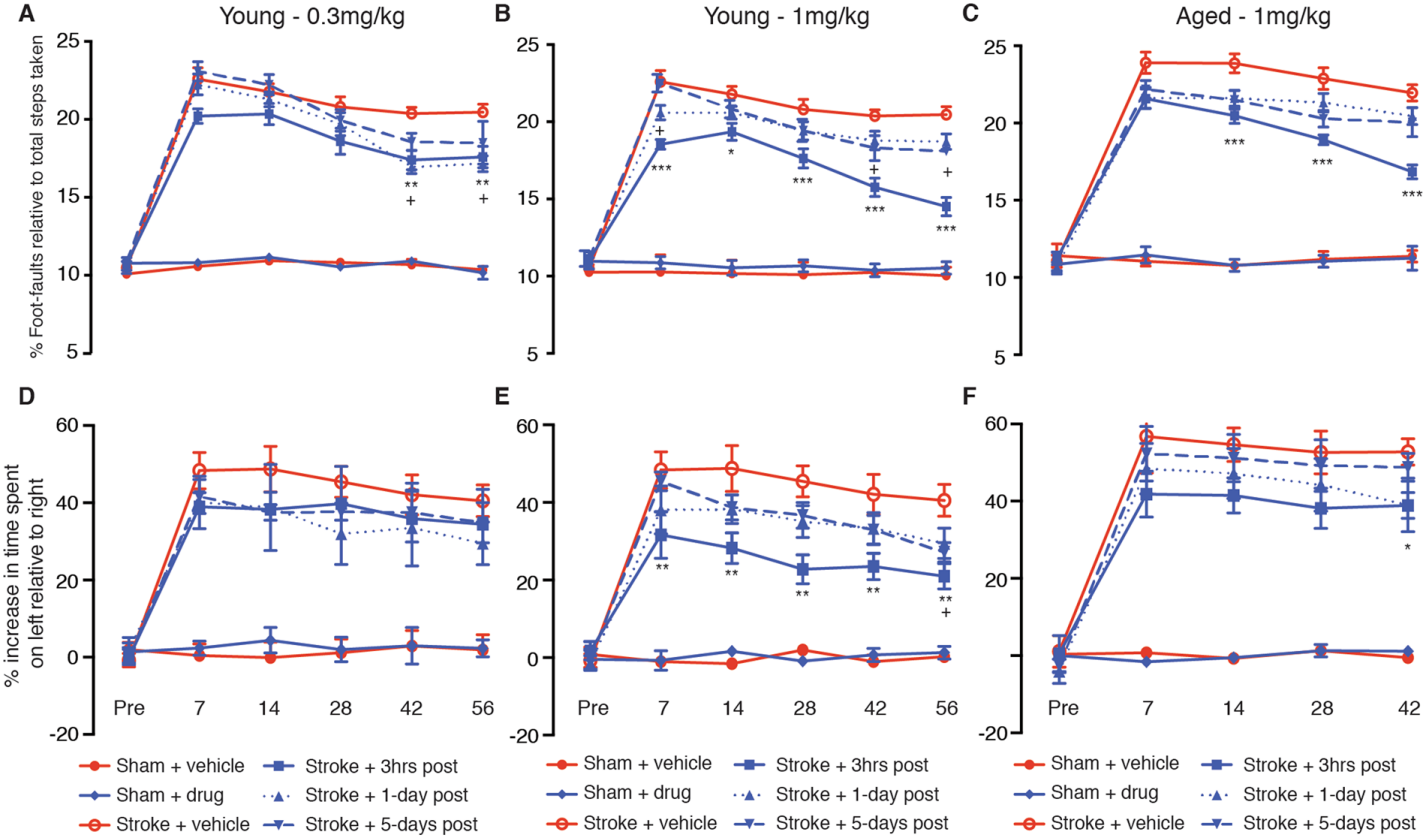
Behavioral recovery was assessed after stroke in young and aged mice. Behavioral recovery in young animals was assessed following 0.3 mg/kg and 1 mg/kg dosing of PEG-IGF-I on both the grid-walking (**A** and **B** respectively) and cylinder/forelimb asymmetry (**D** and **E** respectively) tasks. Behavioral recovery was also assessed in aged animals following 1 mg/kg dosing of PEG-IGF-I on both the grid-walking (**C**) and cylinder/forelimb asymmetry (**F**) tasks. An n = 10 per group were used for these studies. *P < 0.05, **P < 0.01, ***P < 0.001: significance between stroke + vehicle and stroke + PEG-IGF-I-treated from 3 hrs; ^+^P < 0.05: significance between stroke + vehicle and stroke + PEG-IGF-I-treated from 1-day.

High levels of endogenous serum IGF-I during the acute phase of stroke in humans has been shown to correlate with improvements in functional recovery^18^. Additionally, improved behavioral and functional recovery is associated with IGF-I treatment in rodents^28–30^. In order to correlate changes in infarct volume to functional recovery, we next tested the mice behaviourally on both the gridwalking (for forelimb function) and cylinder (for forelimb asymmetry) tasks (n = 10 per group; Fig. 3) out to 8-week post-stroke^22, 24^. Stroke produced an increase in the number of foot-faults in the gridwalking task (Fig. 3A–C), and a decrease in forelimb symmetry in the cylinder task (Fig. 3D–F) from 7 days after stroke. Treatment with 0.3 mg/kg of PEG-IGF-I, 3 hrs post-stroke was found to decrease foot-faults (F(3,216) = 11.69; P < 0.0001: Fig. 3A), but had no effects on cylinder task (F(3,216) = 2.021; P > 0.05: Fig. 3D). A pronounced improvement was observed amongst the mice dosed with 1 mg/kg PEG-IGF-I 3 hrs and 1-day following stroke, on both the gridwalking (F(3,216) = 38.64; P < 0.0001: Fig. 3B) and cylinder tasks (F(3,216) = 16.48; P < 0.0001: Fig. 3E). Dosing 5-days post-stroke with 1 mg/kg showed no functional improvements on either behavioral task. Taken together these results demonstrate that PEG-IGF-I is able to augment functional recovery in stroked mice and again the higher dose (1 mg/kg) administered early after stroke, showed the most pronounced improvements.

We also tested PEG-IGF-I efficacy in the aged (22–24 month old) mice behaviourally on both the gridwalking and cylinder tasks (n = 10 per group; Fig. 3C and F) out to 6-week post-stroke. Treatment with 1 mg/kg of PEG-IGF-I, 3 hrs post-stroke was found to decrease the number of foot-faults from 2-weeks post-stroke (F(3,180) = 21.65; P < 0.0001: Fig. 3C), and a small improvement in the use of the impaired limb in the cylinder task was observed by week 6 post-stroke (F(3,180) = 4.098; P = 0.0076: Fig. 3F). Further treatment starting from either 1 or 5-days post-stroke in the aged mice failed to show any improvement on either the grid-walking or cylinder tasks (Fig. 3C and F).

### PEG-IGF-I modulates GFAP expression

After ischemic stroke astrocytes become activated and increase their expression of GFAP, a hallmark of reactive astrogliosis^31^. Previous studies have shown that IGF-I can actively modulate the expression of GFAP and contribute to the formation of the glial scar^32^. To confirm that similar changes in astrogliosis can occur following treatment with PEG-IGF-I, we investigated changes in GFAP expression within the peri-infarct region following focal ischemia and the corresponding region in the non-lesioned hemisphere.

**Figure 4:**
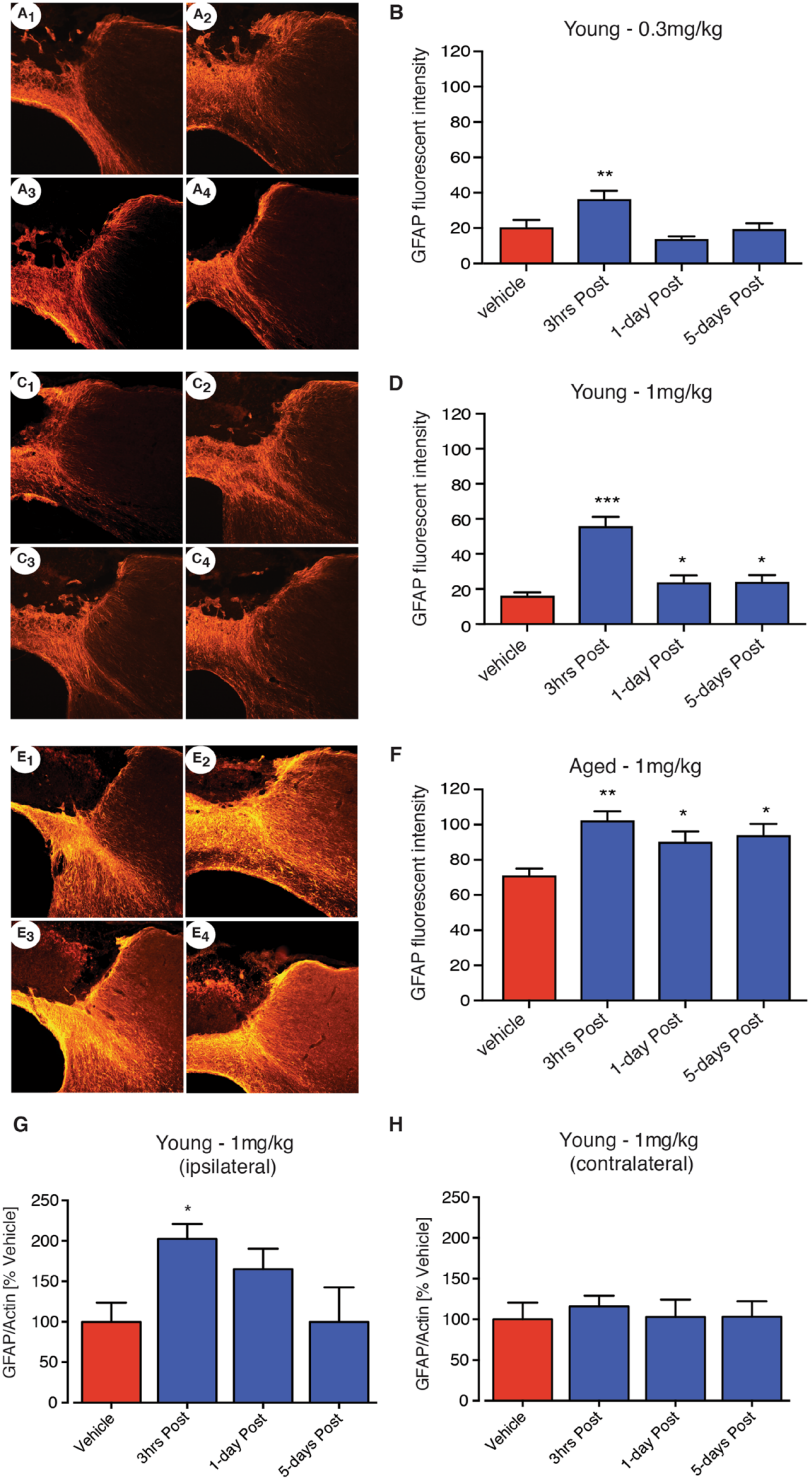
PEG-IGF-I reactive astrogliosis as assessed by GFAP labeling after stroke in both young and aged mice 14-days post-stroke. Representative images of GFAP staining are shown for stroke + vehicle, stroke + PEG-IGF-I from 3 hrs, stroke + PEG-IGF-I from 1-day and stroke + PEG-IGF-I from 5-days post-stroke for young 0.3 mg/kg (**A1**, **A2**, **A3** and **A4** respectively), young 1 mg/kg (**C1**, **C2**, **C3** and **C4** respectively) and aged 1 mg/kg (**E1**, **E2**, **E3** and **E4** respectively) animals. Fluorescent intensity measurements for GFAP were obtained from peri-infarct regions of both young (**B** for 0.3 mg/kg and **D** for 1 mg/kg) and aged (**F** for 1 mg/kg) mice treated with vehicle or PEG-IGF-I. Of note the relative fluorescent intensity measures were approximately 3-fold higher in the aged than young. Western blot analysis was also performed on tissue isolated from both ipsilateral (stroked side: **G**) and contralateral (non-stroked side: **H**) hemispheres of young animals treated with either vehicle or 1 mg/kg PEG-IGF-1. An n = 5 per group were used for these studies. *P < 0.05, **P < 0.01, ***P < 0.001 compared to aged-matched stroke + vehicle-treated controls.

Assessment of GFAP expression by immunohistochemistry in either sham or stroked (vehicle or PEG-IGF-I treatment) young animals showed no difference between treatment groups in the contralateral non-stroked hemisphere (data not shown). Assessment of GFAP expression in the stroked hemisphere revealed an increase in expression indicative of reactive astrogliosis in both young and aged animals (Young: Fig. 4A and C; Aged: Fig. 4E). Young animals treatment with 0.3 mg/kg PEG-IGF-I from 3 hrs post-stroke only resulted in a further increase in GFAP expression (P < 0.01: Fig. 4B). Young animals that were treated with 1 mg/kg PEG-IGF-I revealed a significant increase in reactive astrogliosis when dosed from all three time points, 3 hrs, 1-day and 5-days post-stroke (P < 0.001, P < 0.05, P < 0.05 respectively: Fig. 4D), with the greatest increases in GFAP expression observed following early doing of PEG-IGF-I (3 hrs), which could indicate promotion of an early closure of the glial scar surrounding the stroke.

The relative change in GFAP expression in aged mice as assessed 2-weeks post-stroke following vehicle and 1 mg/kg PEG-IGF-I treated animals was greater than that observed in young. Assessment of PEG-IGF-I-treated animals (1 mg/kg) showed a significant increase in GFAP expression compared to vehicle-treated stroke controls when treatment started from all three time points, 3 hrs, 1-day and 5-days post-stroke (P < 0.01, P < 0.05, P < 0.05 respectively; Fig. 4F), with the greatest increases observed when treatment was started 3 hrs post-stroke.

Western blot analysis was performed on peri-infarct tissue isolated from around the stroke site (ipsilateral) and corresponding brain region on the non-stroked side (contralateral) of young sham and stroke mice. Stroke resulted in an increase in GFAP expression, as assessed 7-days post-stroke following treatment with 1 mg/kg PEG-IGF-I from 3 hrs post-stroke compared to vehicle-treated stroke controls (P < 0.05; Fig. 4G). An increase in GFAP expression was also seen when treatment started from 1-day post-stroke, however this did not reach significance. Assessment of GFAP expression on the contralateral non-stroked side revealed not differences between treatment groups or time periods (Fig. 4H).

### PEG-IGF-I treatment increases neurogenesis

**Figure 5:**
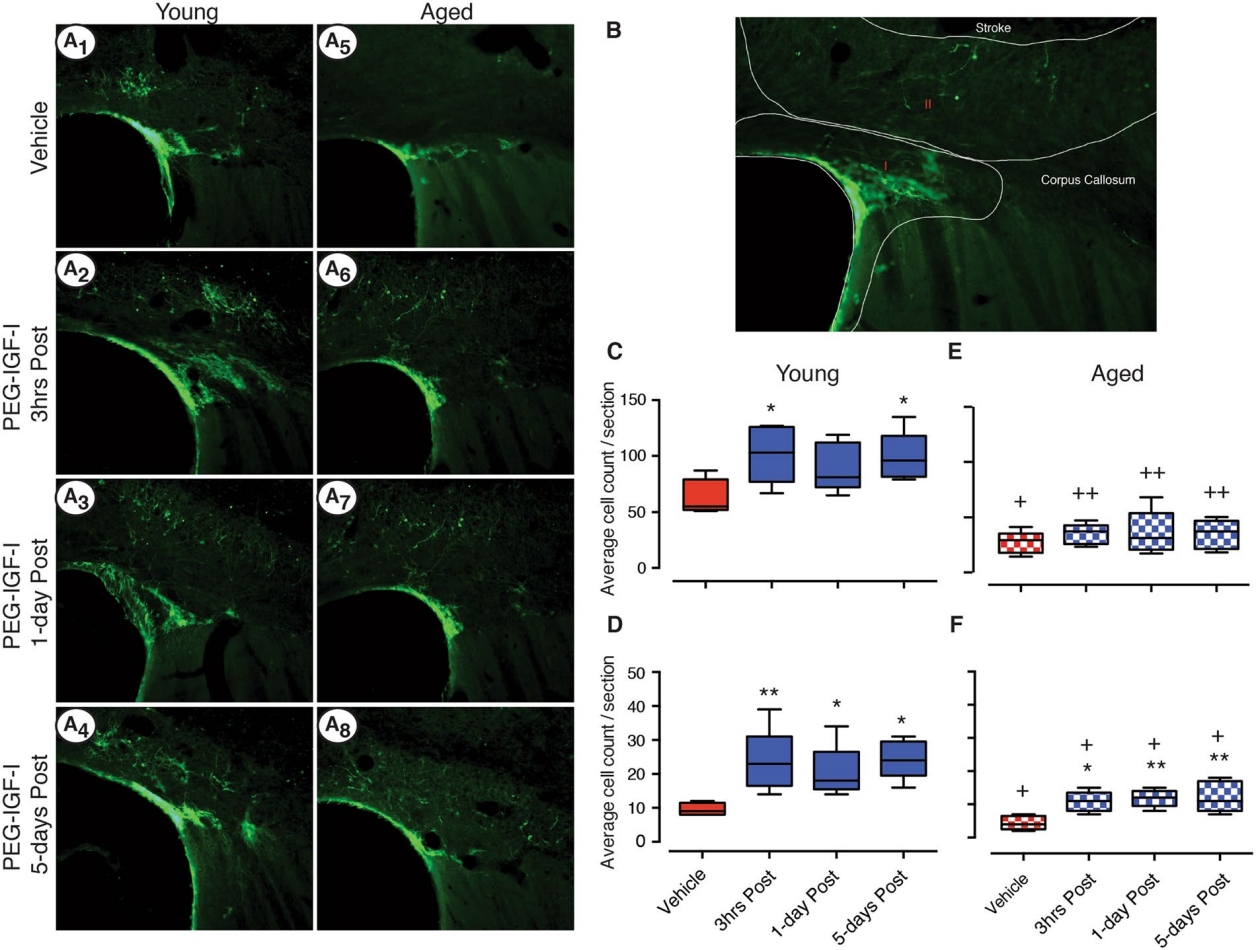
PEG-IGF-I stimulates the migration of neuroblasts *in vivo* after stroke in both young and aged mice 14-days post-stroke. Representative images of DCX + cells migrating from the lateral ventricle are shown for stroke + vehicle, stroke + 1 mg/kg PEG-IGF-I from 3 hrs, stroke + 1 mg/kg PEG-IGF-I from 1-day and stroke + 1 mg/kg PEG-IGF-I from 5-days post-stroke for young (**A1**, **A2**, **A3** and **A4** respectively) and aged (**A5**, **A6**, **A7** and **A8** respectively) animals. Panel B shows a sample photomicrograph illustrating the regions where cells were counted. Region A is next to the lateral ventricle including the bottom half of the corpus callosum, whereas region B is counts that include the top half of the corpus callosum and surrounding the stroke cavity. Box and whisker plots show the average cell counts from three section per animal of n = 5 animals per treatment group for each of the two regions (**C** and **D** for regions I & II respectively) for young and (**E** and **F** for regions I & II respectively) for aged. *P < 0.05, **P < 0.01 compared to stroke + vehicle treated controls controls; ^+^P < 0.05, ^++^P < 0.01 compared to the equivalent young treated controls.

Promoting neural regeneration after cerebral infarction has emerged as a potential approach for the treatment of stroke. IGF-I possesses both neurotrophic and angiogenic properties, therefore we next investigated whether PEG-IGF-I might stimulate an increase in neurogenesis, a mechanism associated with neurorepair. We performed doublecortin (a cell marker expressed by immature neurons emerging and migrating from the SVZ reflecting neurogenesis)^33, 34^ immunofluorescence on coronal brain sections generated 14-days post-stroke from young and aged animals that underwent photothrombosis and received either vehicle or PEG-IGF-I treatment starting 3 hrs, 1-day or 5-days post-stroke (Fig. 5A and B).

Assessment of young mice that received PEG-IGF-I treatment from either 3 hrs or 5-days post-stroke revealed a significant increase in the number of doublecortin positive cells that line the lateral ventricle (Region A shown in Fig. 5B: P < 0.05: Fig. 5C) as well as a significant increase in the number that migrate to the site of the stroke (Region B shown in Fig. 5B: P < 0.01, P < 0.05 respectively: Fig. 5D). Animals that received treatment from 1-day post-stroke showed a small increase in neurogenesis, with significance only observed in the number of cells that migrated to the site of injury (P < 0.05: Fig. 5D).

As post-stroke neurogenesis is known to be impaired in aged animals, we sought to expand our findings by assessing PEG-IGF-I treatment on post-stroke neurogenesis in in aged (22–24 month old) animals. In corroboration with prior studies, we also observed a significant decrease in the number of doublecortin positive cells around the lateral ventricle (Fig. 5E), and those that migrated to the site of injury (Fig. 5F). Treatment with PEG-IGF-I starting at 3 hrs, 1-day or 5-days post-stroke had no overall effect on neurogenesis as indicated by unchanged numbers of neuroblasts in close proximity to the lateral ventricle (Fig. 5E). Interestingly, a small but significant increase in the number of neuroblasts that migrated to the site of injury was noted following PEG-IGF-I treatment starting at 3 hrs, 1-day and 5-days post-stroke (P < 0.05, P < 0.01, P < 0.01 respectively: Fig. 5F).

### PEG-IGF-I increased the presynaptic marker synaptophysin

**Figure 6:**
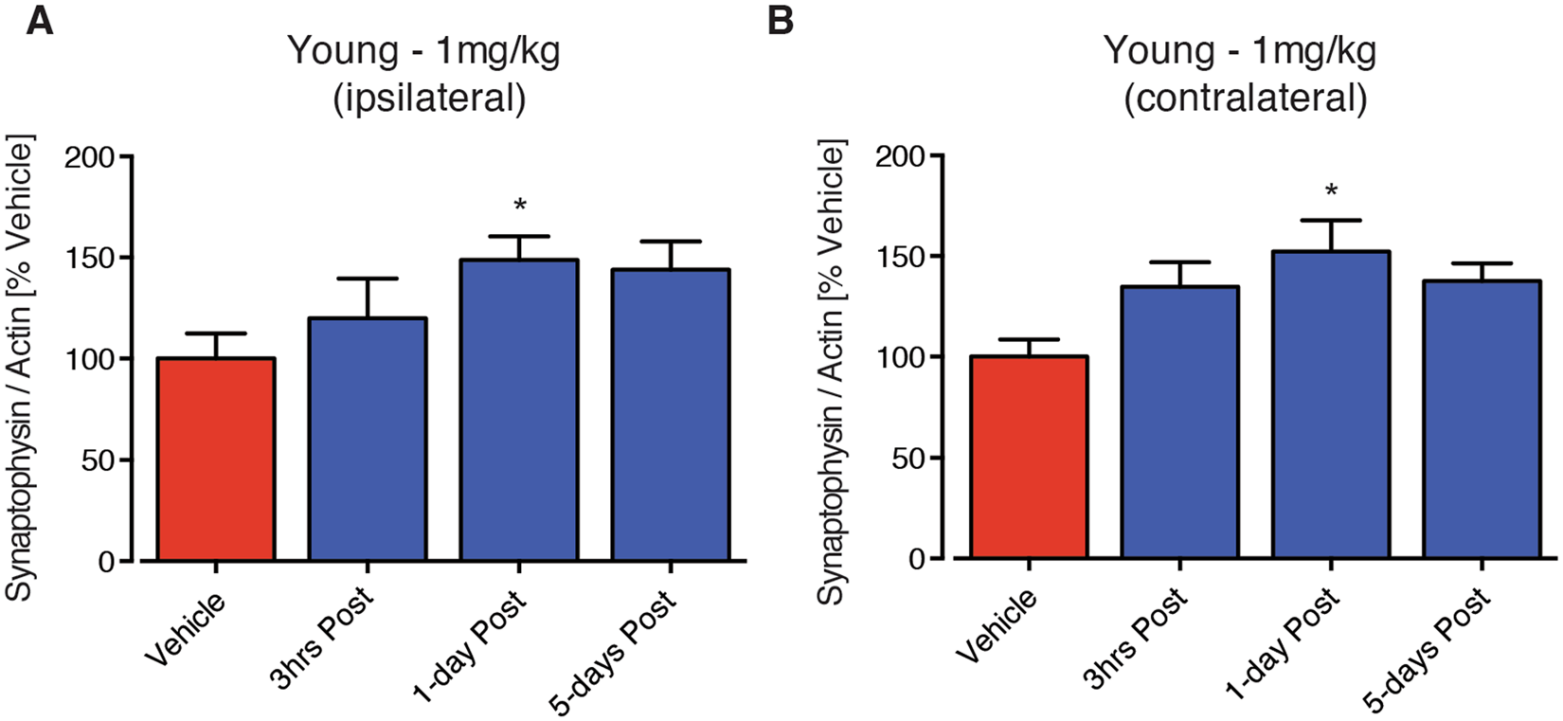
PEG-IGF-I increases the expression of the presynaptic protein, synaptophysin 7-days post-stroke both ipsilateral (stroke side: **A**) and contralateral (non-stroked side: **B**) to the lesion. An n = 5 per group were used for these studies. *P < 0.05, compared to aged-matched stroke + vehicle-treated controls.

Synaptogeneiss is a critical process for brain repair and for regaining lost functions. IGF-I has been previously reported to lay a role in increasing synaptogenesis. In order to assess the effects of PEG-IGF-I treatment on changes in synaptogeneis, we investigated the presynaptic marker synaptophysin using Western blot analysis on peri-infarct tissue isolated from around the stroke site (ipsilateral: Fig. 6A) and corresponding brain region on the non-stroked side (contralateral: Fig. 6B) of young sham and stroke mice. Synaptophysin expression was assessed 7-days post-stroke following treatment with 1 mg/kg PEG-IGF-I in young animals and shown to be elevated on both ipsilateral (stroke side) and contralateral (non-stroked side) hemispheres following treatment starting from 3 hrs, 1-day and 5-days post-stroke, although results were only significant for the 1-day post-stroke time period. These data confirm that PEG-IGF-I is having a small yet significant effect on synaptogenesis and neurorepair. Interestingly this data also highlights that specific critical periods after stroke exist, with a therapeutic window opening after the stroke-induced change has occurred.

### PEG-IGF-I increased axonal sprouting

**Figure 7:**
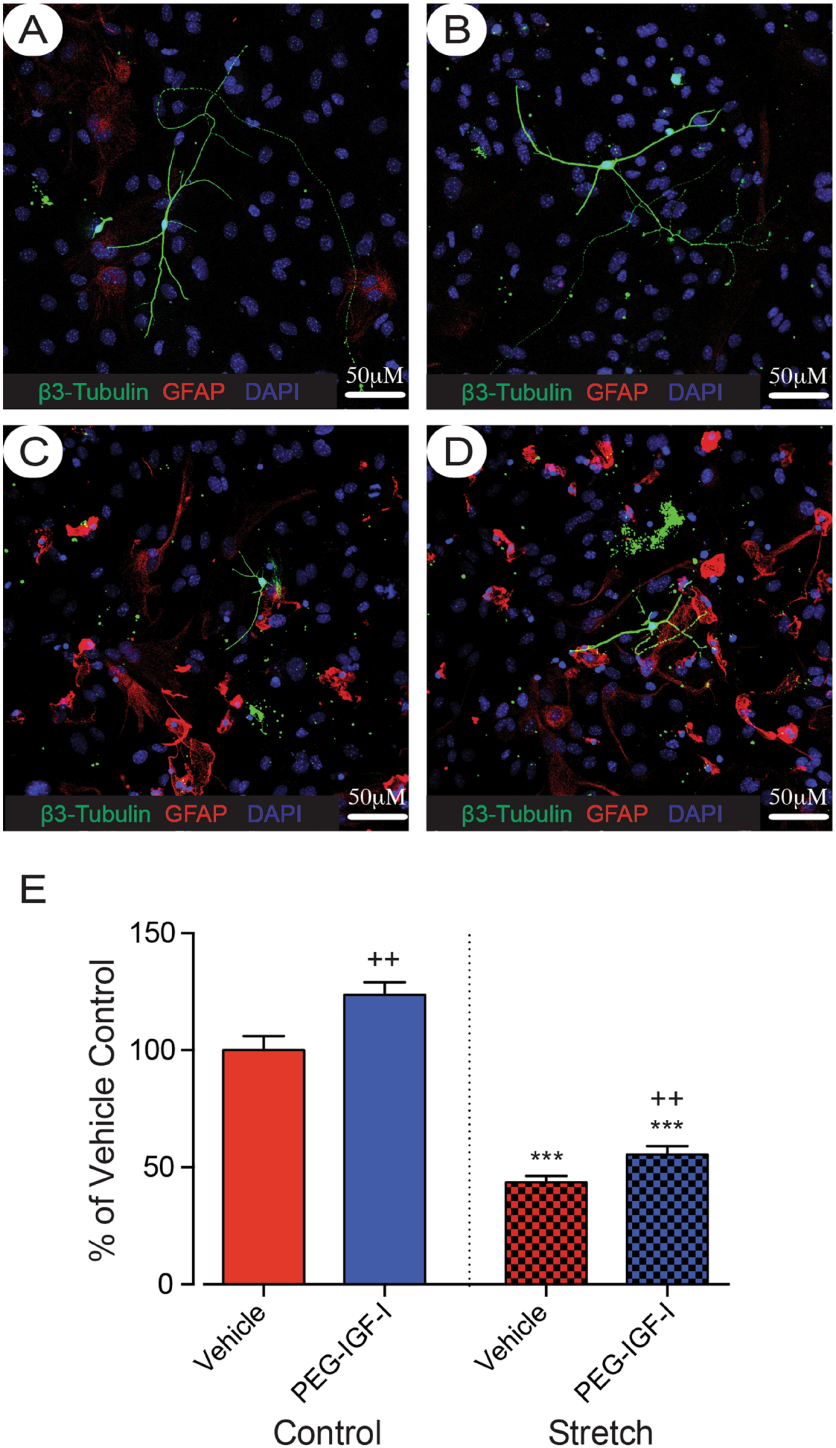
PEG-IGF-I increases neurite outgrowth of cortical neurons plated on top of stretched astrocytes. Representative images of individual neurons are shown for control + vehicle (**A**), control + 100 ng/mL PEG-IGF-I (**B**), stretch + vehicle (**C**), and stretch + 100 ng/mL PEG-IGF-I (**D**), scale bar represents 50 μm. Reactive astrocytes are shown in red (GFAP), neurons in green (β3 tubulin) and the nuclear counter label in blue. Quantification of total neurite lengths (**E**) reveals that 24 hrs treatment with 100 ng/mL PEG-IGF-I increases neurite outgrowth of neurons plated on top of stretched astrocytes but not control astrocytes. Data is expressed as mean ± SEM from 3 independent co-cultures, each performed in triplicate (45 neurons per condition were analysed). ***P < 0.001 compared with vehicle-treated non-stretched controls; ^++^P < 0.001 compared with vehicle-treated stretch and non-stretch controls.

Reactive astrogliosis is known to inhibit neurite outgrowth^7, 8^. Therefore, we next investigated whether activation of the IGF-I-signaling pathway, which is also involved in developmental neurite growth, might be able to overcome this injury- and astrogliosis-induced neurite growth impairment. In order to assess the effects that reactive astrogliosis had on cortical neuron outgrowth and the therapeutic effects of PEG-IGF-I on this interaction, we aimed to confirm the data in an *in vitro* model of reactive astrogliosis. This model consisted of mechanically traumatizing mature astrocytes cultured on deformable plates^7, 35^, which results in morphological changes and up-regulation of GFAP expression in astrocytes (red immunostaining shown in Fig. 7C and D).

Neurites were quantified 24-hours after culturing cortical neurons on top of either control (non-stretched) or reactive astrocytes. Neurons plated on control (non-stretched) astrocytes were able to extend long neurites (Fig. 7A), whereas, neurons that were plated on top of stretched reactive astrocytes had significantly reduced neurite growth (P < 0.001; Fig. 7C and E). Addition of 100 ng/ml PEG-IGF-I to the media increased the total neurite length of neurons plated on both non-stretched and stretched reactive astrocytes (P < 0.01; Fig. 7B and D respectively) when compared with vehicle-treated neuron controls. Thus, from these results it appears that PEG-IGF-I is able to augment axonal sprouting in the presence of reactive astrogliosis and potentially contribute in the regrowth of connections after stroke and enhance recovery.

## Discussion

Stroke remains one of the leading causes of death and long-term impairment. Despite decades of research, there are limited treatment strategies to either minimize the extent of cellular damage or enhance recovery of lost functions. IGF-I has been shown to have a number of key roles within the brain, including an important part in normal brain development, promoting neuronal growth, cellular proliferation and differentiation^11^. Exogenous IGF-I has been shown to protect against ischemic brain injury including facilitating the growth of new connections and contributing to regenerative processes after stroke^10, 12–15^. One of the major limitations of exogenous recombinant human IGF-I (rh-IGF-I) treatment is its relatively short half-life and side effects such as hypoglycaemia, and suppression of Growth Hormone release^36, 37^. The aim of the current study was to assess the therapeutic potential of PEG-IGF-I for stroke, which we show following a single i.p. injection results in sustained serum levels even 48 hrs after dosing, similar to previous reports^19^.

We report that early (3 hrs-1-day) treatment with PEG-IGF-I affords the greatest therapeutic potential as evidenced by a decrease in infarct volume and improved motor functional recovery in both young and aged mice. Whilst delaying treatment until 5-days post-stroke resulted in an increase in neurogenesis, this did not translate into improved functional recovery. In support of these findings, a recent study showed that local delivery of IGF-I in a hydrogel injected into the stroke cavity, providing sustained IGF-I release to the peri-infarct cortex for a period of up to 4-weeks, does not induce changes in cortical connections, although delivery of the IGF-I antagonist JB1 resulted in a decrease in the distribution of cortical connections compared to vehicle treated stroke controls^9^. This suggests a yet unknown role of IGF-I signaling after stroke by supporting maintenance of cortical connections. In addition, the study by Li and colleagues supports the hypothesis that IGF-I is protective after stroke^16, 17^, as JB1 administration increased cell death within the peri-infarct cortex of stroked mice^9^.

With the advances and positive outcome of recent clinical trials using intra-arterial endovascular thrombectomy^38–41^, this opens up the avenue for combined pharmacotherapy^42^. Many compounds have been trialed in preclinical studies, however, they have failed to translate into clinical use due to their inability to cross the blood-brain-barrier, or un-wanted side-effects due to high doses required. Combining intra-arterial drug administration as an adjunct therapy with endovascular thrombectomy provides timely (i.e. early) and selective targeting of compounds to the cerebral vasculature. As we report that early (3 hrs-1-day) treatment with PEG-IGF-I affords the greatest protection, administration of PEG-IGF-I as an adjunct with mechanical recanalization may afford greater protection and functional improvements.

### Effects of PEG-IGF-I treatment on neurogenesis

Our results show that treatment with PEG-IGF-I resulted in an increase in neurogenesis. These findings are consistent with previous publications showing that after ischemic injury, various growth factors including IGF-I can stimulate an increase in neurogenesis^43, 44^. For instance, infusion of IGF-I protein via osmotic minipumps increases stroke-induced progenitor cell proliferation in hypertensive rats^43^. Further, recent data shows that transfection using an AAV-IGF-I increases the number of neuronal progenitor cells in the SVZ zone of infarcted hemispheres compared with AAV-GFP treated stroke controls^44^. Consistent with these data, we show that treatment with PEG-IGF-I stimulates an increase in doublecortin expression and migration from the subventricular zone. Further we show that PEG-IGF-I can increase the expression of the presynaptic protein, synaptophysin, which is a component of synaptic vesicles that helps determine the efficacy and strength of synapses and inturn synaptic plasticity and neural repair. Taken together these data suggest that PEG-IGF-I could be a viable treatment option to enhance recovery after stroke via an increase in neuroblasts and outgrowth of neurites, which can offer growth factor support to the peri-infarct tissue. However, further studies are required to confirm this hypothesis as neurogenesis was also present in animals treated 5-days post-stroke that showed no improvement in motor function. It is possible that the level of neurogenesis present is not enough to overcome the stroke-induced barriers and aid in improving motor functional recovery, raising the question what is the desired level of neurogenesis to achieve functional recovery. This notion of needing to reach a threshold in order to achieve a response is not a new one and has been shown to be critical for the growth factor Brain Derived Neurotrophic Factor to be effective at improving function, including in aged rodents^45, 46^. Although not measured, one would speculate that in addition to neurogenesis, angiogenesis is also altered. Prior studies have reported that the processes of angiogenesis and neurogenesis are temporally and spatially-coupled^47^, with blood vessels and neuronal fibers developing side-by-side and guiding each other to migrate to the target area.

### Effects of PEG-IGF-I treatment on reactive astrogliosis

The formation of the glial scar and expansion of reactive astrogliosis into surrounding peri-infarct territories plays an essential role after stroke. While it can serve to confine the stroke and limit the spread of cellular damage into the penumbra and/or regions of healthy tissue if promoted acutely, it can also impede recovery by serving as a physical barrier to neurorepair during the chronic phase^48^. PEG-IGF-I appears to act on post-stroke reactive astrogliosis when given early (3 hrs post-stroke) by increasing GFAP expression, a hallmark of reactive astrogliosis^31^. This process is likely helping to limit the extent of neuronal cell death, as evidenced by a decrease in infarct volume.

GFAP is upregulated as early as 1-day after injury and the number of reactive astrocytes is significantly increased around the lesion site from 3–5 days post-injury^49^. Our most pronounced effects were observed when treatment was started 3 hrs post-stroke, which is in line with astrocyte processes increasing within hours of brain injury^50^. Taken together these results imply that PEG-IGF-I may augment the protective activity of astrocytes for an acute period after insult and mitigate detrimental inflammatory and immunological processes in the lesion site, thus supporting functional recovery. These are studies that are ongoing in the laboratory in order to confirm these hypotheses.

### Effects of PEG-IGF-I treatment of axonal sprouting

Prior studies have shown that neurons can sprout new connections and brain regions can re-map following both peripheral and central nerve injury^51–53^. Stroke induces cortical neurons to switch into a growth mode: to sprout new axons, elongate and form new patterns of connections^54, 55^. This process may represent a re-activation of a developmental cellular program, or it may involve unique adult regeneration-associated genes^56, 57^, including IGF-I signalling^9^. The processes associated with axonal sprouting are highly influenced by changes in the cellular environment^58^. While astrocytes usually support neurons and facilitate neuronal maturation under normal conditions, when they become reactive as a consequence of cellular damage, they can inhibit axonal sprouting and impair functional recovery. Overcoming this astrocytic inhibition is a plausible therapeutic approach for improving recovery of brain function following injuries such as stroke^59^. Here we focussed on the role of IGF-I signalling in post-stroke CNS repair, given its role in CNS development^11^. We show that treatment with PEG-IGF-I increases neurite outgrowth from post-natal cortical neurons grown on top of reactive astrocytes. Further, we also report that GAP43, a putative marker of axonal growth was elevated following PEG-IGF-I treatment 3 hrs post-stroke *in vivo*. These data are similar to what has been reported previously, whereby targeting specific signalling pathways, such as Ephrin-A5 or sonic hedgehog, can stimulate axonal sprouting and facilitate post-stroke recovery^7, 8^. However, unlike these previous studies where reactive astrogliosis is dampened, PEG-IGF-I stimulated axonal sprouting also results in an increase in reactive astrogliosis as indicated by an increase in GFAP expression. Whilst the current dogma is that reactive astrogliosis impairs axonal sprouting and functional recovery post-injury, we show here that this does not always hold true. In support of these findings is a recent publication in ‘Nature’ highlighting that the formation of the astrocytic scar aids rather than prevents CNS axonal regeneration^60^. In further support with these findings, we show that synaptophysin in elevated following treatment with PEG-IGF-I, indicating improved synaptic plasticity.

### Summary

We report that dosing with PEG-IGF-I results in a sustained increase in serum IGF-I levels, which in turn offers better therapeutic dosing potential than conventional IGF-I treatment. As a result, PEG-IGF-I demonstrates good capabilities in alleviating damage to neural tissue and containment of the infarct site. Further, PEG-IGF-I can enhance functional recovery post-stroke, in part via an increase in axonal sprouting, improved synaptic plasticity, altered GFAP expression and increased neuroblast migration in both young and aged mice, mechanisms that are all required to aid rehabilitation of stroke victims. In conclusion, these findings indicate that early, 3 hrs-1-day and not delayed, 5-days post-stroke PEG-IGF-I treatment affords the greatest benefits in facilitating an improvement in motor function and could be a potential candidate for therapeutic use in a clinical setting for stroke patients. Interestingly, early PEG-IGF-I treatment enhances reactive astrogliosis and stimulates axonal sprouting and synaptic plasticity, going against the common dogma that reactive astrogliosis impairs recovery, and highlights a need for a better understanding for the role of reactive astrogliosis under pathological conditions.

## Materials and Methods

### Animals

All procedures described in this study were approved by the University of Otago, Animal Ethics Committee in in accordance with the ARRIVE guidelines. Young (2–3 month old) and aged (22–24 month old) male mice (C57BL/6J) were housed under a 12 hr light, 12 hr dark cycle (lights on 0700 hrs) with *ad libitum* access to food and water. All mice were assigned to experimental groups in a randomized manner, with assessments undertaken in a blinded fashion. For these studies a total of 316 mice were used, 208 young and 108 aged for the *in vivo* studies and 6 litter for the *in vitro* studies. All samples sizes for the various assessment parameters were calculated based on our own prior studies and use of sample size calculators^7, 8, 24, 45, 61^.

### *In vivo* experiments

#### Photothrombosis model of focal ischemia

Focal stroke was induced in the left hemisphere using the photothrombosis method in either young adult (2–3 month old, 25–27 g) or aged (22–24 month old, 30–34 g) male C57BL/6J mice^22–24^. In brief, under isoflurane anesthesia (2–2.5% in medical O_2_) mice were placed in a stereotaxic apparatus, the skull exposed and a cold light source (KL1500 LCD, Zeiss) attached to a 20x objective giving a 2 mm diameter illumination positioned 1.5 mm lateral from Bregma. 0.2 mL of Rose Bengal solution (Sigma; 10 g/L in normal saline) was administered intraperitoneally (i.p.). After 5 min the brain was illuminated for 17 min. Body temperature was maintained at 36.9 ± 0.3 °C with a heating pad (Harvard apparatus) throughout surgical procedures. After a brief recovery period animals were returned to their normal housing conditions.

#### Source of PEG-IGF-I

PEG-IGF-I was generated in house at Hoffmann La-Roche as previously described^19^. Briefly, PEG-IGF-I is recombinant human IGF-I mutated at amino acids 27 and 65 (each Lysine => Arginine) and modified by addition of PEG of 40 kDa at lysine 68. The production process has been described^19^, and it was stored in 20 mM NaAcetate, 140 mM NaCl, pH 5, at 1 mg/ml (protein amount of the drug). For *in vivo* experiments, stocks were directly diluted PBS at the required concentrations.

#### *In vivo* drug dosing

PEG-IGF-I (0.3 mg/kg or 1 mg/kg) or PBS-vehicle was given via i.p. injection, at different times following stroke and twice weekly thereafter. All dosing was given by an independent experimenter not undertaking any of the histological, biochemical or behavioural assessments to randomise the animals and minimise bias, with dosing starting either 3 hrs, 1-day, or 5-days after stroke. For assessment of serum concentration, cohorts of animals were sacrificed and bloods collected at 0, 6, 24 or 48 hrs following a single i.p. injection of either 0.3 or 1 mg/kg PEG-IGF-I.

#### ELISA detection of serum PEG-IGF-I

PEG-IGF-I was detected in serum as previously described^19^. Briefly, concentrations were determined after an acid/neutralization step to dissociate PEG-IGF-I/protein complexes, using a biotinylated mouse-anti-PEG antibody on streptavidin-coated microtiter wells and digoxigenylated rhIGFBP-4 along with sheep anti-digoxigenin Fab fragments conjugated to horseradish peroxidase for detection. ABTS was used as substrate and absorbance measured at 405/490 nm^19^.

#### Behavioral assessment

Animals were tested once on both the gridwalking and cylinder tasks, one week prior to surgery to establish baseline performance levels. For all of the studies, animals were tested on weeks 1, 2, 4, 6 and 8 post-stroke at approximately the same time each day at the end of their dark cycle. All behaviors were scored by observers who were blind to the treatment group of the animals in the study as previously described^22–24^. Ten animals per group were assessed on all behavioral tasks.

#### Immunohistochemical and histological assessments

At 14-days post-stroke, young (2–3 month old) and aged (22–24 month old) vehicle and PEG-IGF-I treated animals were anesthetized, transcardially perfused with 4% paraformaldehyde and brains extracted and processed histologically using cresyl violet staining in order to quantify infarct volume as previously described, using 30 μm thick coronal sections collected using a sliding microtome^22–24^.

Immunocytochemistry was performed for GFAP and doublecortin to assess the effects of PEG-IGF-I on glial scar formation and neurogenesis respectively. Briefly, sections were washed thoroughly in TBS, blocked in 5% donkey serum, and incubated for 48 hrs at 4 °C in either the polyclonal chicken anti-GFAP (dilution 1:2000; Millipore, USA) or in the polyclonal guinea pig anti-doublecortin (1:500: Cat# AB2253; Millipore) primary antibodies, diluted in TBS containing 0.3% Triton X-100 and 0.25% bovine serum albumin (hereafter referred to as incubation solution) containing 2% normal donkey serum. Sections were then washed three times in TBS (10 min per wash) before being incubated in either the donkey anti-chicken 549 secondary antibody (1:400; Jackson Immunoresearch, USA) or the donkey anti-guinea pig 488 DyLight (1:400; Sapphire Bioscience, USA) secondary antibody at a dilution of 1:400 in incubation solution for 90 min at room temperature. After subsequent washing in TBS, sections were mounted onto gelatin-coated glass slides, air-dried, passed sequentially through alcohols (50%, 70%, 95% & 100%) before being passed through xylene and then coverslipped using DPX mounting solution.

Images of the glial scar (400 um from the stroke border) encompassing what is known as the peri-infarct region, were taken on an Olympus BX51 microscope and fluorescent intensity measures taken using ImageJ analysis. Fluorescent intensity measurements of the scar were normalised to background reading on the contralateral hemisphere and an average fluorescence obtained using 3-sections from n = 5 animals. An observer blind as to the treatment group took the images and fluorescent intensity measurements. Quantification of doublecortin staining was carried out by counting all doublecortin positive cells from three sections per animal from either (I) the region surrounding/next to the lateral ventricle, or (II) the region extending from the corpus callosum into the cortex and surrounding the stroke cavity (see Fig. 5).

#### Western blotting

Tissue was collected from around the stroke site from stroke + vehicle, stroke + PEG-IGF-I and naïve control groups 7-days post stroke. Cortical tissue was dissected in a 1 mm radius around the stroke infarct core, including the core itself, and flash frozen on dry ice. Equal volumes of tissue were homogenized in 100 ml homogenization buffer containing; Complete Protease Inhibitor Tablet (Invitrogen, Carlsbad, CA), 1 mM phenylmethylsulfonylfluride, 50 mM Tris-HCl, 5 mM EDTA, 10 mM EGTA, 1% Triton X-100) for approximately 1 min. Tissue and homogenization buffer were incubated on ice for 30 min, followed by a 5 min spin at 14,000 *g* at 4 °C and supernatants were stored at −80 °C. Total protein concentration was determined by DC Kit (Bio-Rad Laboratories, Inc.) and 10 μg of total protein was mixed with the same volume of sample buffer and incubated at 95 °C for 5 min and loaded into 10% PAGE gels (Mini-protean TGX precast gels, Bio-Rad Laboratories, Inc.). Proteins were transferred to PVDF membrane using Trans-Blot Turbo Transfer System (Bio-Rad Laboratories, Inc.). Blots were blocked for 30 min using Odyssey Blocking Buffer (LI-COR Biosciences, Lincoln, NE, USA) and incubated with primary antibody for 2 hrs at room temperature. The primary antibodies used were: mouse anti-Synaptopysin (Sigma Cat. No. # S5768: 1:2000), goat anti-Actin (SantaCruz Cat. No. # sc-1615: 1:20,000), rabbit anti-GFAP (Dako Cat. No. # Z0334: 1:1,000); secondary antibodies used were: donkey anti-goat IRDye800 (Rockland Cat. No. # 605-732-125: 1:2,000) and donkey anti-rabbit IRDye700 (Rockland Cat. No. # 611-730-127: 1:10,000). Immunoreactivity was captured and quantified using the Odyssey Infrared imaging system (LI-COR Biosciences).

### *In vitro* experiments

#### Cortical astrocyte cultures and stretch trauma model

Astrocyte stretch trauma was performed as previously reported^7, 35^ with minor modifications^8^. Astrocyte cultures were prepared from cortices of postnatal day (P)1–2 mice. The cortices were isolated, incubated for 10 min at 37 °C with 0.25% trypsin (Life Technologies Carlsbad, California, United States). Trypsin was inactivated with DMEM/F12 (Life Technologies) + 10% fetal bovine serum (FBS, New Zealand origin; Life Technologies). Cells were then dissociated by gentle pipetting in DMEM/F12, supplemented with 1% Penicillin/Streptomicin (Life Technologies) and 10% FBS, and plated (2 cortices per flask) in 75 mm^2^ flasks (Corning). The medium was changed every 3–4 days. After 10–12 days, cultures were shaken for 24–36 hours (180 rpm) and treated with 10 mM leucin methyl ester (Sigma-Aldrich, Castle Hill, Australia) for 12 hrs to remove oligodendrocyte precursors and microglia. Secondary astrocyte cultures were then established by trypsinizing the primary cultures, and subplating 300,000 cells onto 35 mm-diameter deformable membrane wells coated with collagen I (Bioflex 6 well plates, Flexcell International, McKeesport, PA, USA). After 1-week the percentage of FBS in the medium was reduced to 5%, and after an additional 1–2 weeks the serum was reduced to 0.5% for 48 hrs and then the cells were grown in the absence of serum. Six-hours after the removal of serum, the cells were mechanically traumatized using an abrupt pressure pulse with a pneumatic device (Flexcell FX-4000 Strain Unit, Flexcell International, McKeesport, PA, USA) programmed to produce a maximal elongation of 23% (130 ms, triangular stretch).

#### Post-natal cortical neuronal cultures

Twenty-four hours after the mechanical trauma, cortical neurons were isolated from P5–6 mice and plated on top of the reactive astrocytes as previously reported^7, 8, 35^. Cortices were dissected in Hibernate medium (Life Technologies) and incubated for 30 min at 30 °C with 2 mg/ml papain (Sigma-Aldrich, Australia). After enzymatic digestion, cells were dissociated by gentle pipetting and the cellular suspension was purified by Optiprep (Sigma-Aldrich) gradient to isolate neurons from the other cells as previously reported^7, 8, 35^. Dissociated cortical neurons were plated at a density of 20,000/well on top of pre-prepared astrocyte cultures.

All pharmacological treatments were performed for 24 hrs in medium containing 0% FBS. PEG-IGF-I was diluted in PBS and used at a final concentration of 100 ng/mL.

#### Immunocytochemistry

Cultures were fixed with 4% paraformaldehyde (PFA) for 10 min at room temperature (RT). Cultures were incubated with phosphate buffered saline (PBS) containing 0.2% Triton X-100 (Sigma-Aldrich) for 10 min at RT and then with blocking solution (PBS containing 2% bovine serum albumin (Sigma-Aldrich)) for 30 min at RT with gentle shaking. Subsequently, cultures were incubated for 2–3 hrs at RT with mouse anti-β3 tubulin (TuJ1 clone, 1:2000, R&D Systems, Inc., Minneapolis, USA) and chicken anti-GFAP (1:5000, Millipore) antibodies. After washing (3 × 10 min in PBS at RT) cultures were incubated for 2 hrs at RT with 488 and 549 DyLight dyes conjugated to affinity-purified secondary antibodies (1:1000, Jackson ImmunoResearch). Hoechst 33258 (0.4 μg/ml, Sigma-Aldrich) was used for nuclear counterstaining.

#### Neurite outgrowth Assay

For each well, all neurons (around 15–30 TuJ1-positive cells) in a randomly selected 110 mm^2^ area were photographed digitally using an inverted fluorescence microscope with a 20x objective (IX71 Olympus, Tokyo, Japan). Neurite outgrowth was quantified using ImageJ (1.45 S version, NIH). The total neurite number and the longest neurite lengths were measured. The analysis was performed by analyzing at least 45 neurons per condition across three independent co-culture preparations performed in triplicate.

#### Statistical Analysis

Statistical significance was assessed by one-way analysis of variance (ANOVA) followed by the Tukey post-hoc test (GraphPad Software, San Diego, CA) or by Student’s t-test when only two groups were compared. Data shown in the figures are the results of at least three independent experiments. Fluorescent intensity measures were analyzed using Student t-tests. Data are represented as mean ± SEM. P < 0.05 was considered significant for all analyses.
